# Small molecule profiling to define synergistic EGFR inhibitor combinations in head and neck squamous cell carcinoma

**DOI:** 10.1002/hed.27018

**Published:** 2022-02-27

**Authors:** Nicole L. Michmerhuizen, Megan L. Ludwig, Andrew C. Birkeland, Sai Nimmagadda, Jingyi Zhai, Jiayu Wang, Brittany M. Jewell, Dylan Genouw, Lindsay Remer, Daniel Kim, Susan K. Foltin, Apurva Bhangale, Aditi Kulkarni, Carol R. Bradford, Paul L. Swiecicki, Thomas E. Carey, Hui Jiang, J. Chad Brenner

**Affiliations:** ^1^ Department of Pharmacology University of Michigan Medical School Ann Arbor Michigan USA; ^2^ Department of Otolaryngology—Head and Neck Surgery University of Michigan Medical School Ann Arbor Michigan USA; ^3^ Program in Cellular and Molecular Biology University of Michigan Medical School Ann Arbor Michigan USA; ^4^ Department of Biostatistics University of Michigan School of Public Health Ann Arbor Michigan USA; ^5^ Department of Hematology and Oncology University of Michigan Medical School Ann Arbor Michigan USA; ^6^ Rogel Cancer Center University of Michigan Medical School Ann Arbor Michigan USA

## Abstract

**Background:**

Head and neck squamous cell carcinoma (HNSCC) is a debilitating disease with poor survival. Although epidermal growth factor receptor (EGFR)‐targeting antibody cetuximab improves survival in some settings, responses are limited suggesting that alternative approaches are needed.

**Methods:**

We performed a high throughput drug screen to identify EGFR inhibitor‐based synergistic combinations of clinically advanced inhibitors in models resistant to EGFR inhibitor monotherapies, and then performed downstream validation experiments on prioritized synergistic combinations.

**Results:**

From our screen, we re‐discovered known synergistic EGFR inhibitor combinations with FGFR or IGF‐1R inhibitors that were broadly effective and also discovered novel synergistic combinations with XIAP inhibitor and DNMT inhibitors that were effective in only a subset of models.

**Conclusions:**

Conceptually, our data identify novel synergistic combinations that warrant evaluation in future studies, and suggest that some combinations, although highly synergistic, will require parallel companion diagnostic development to be effectively advanced in patients.

**Abbreviations**
ALK: anaplastic lymphoma kinase
AURKA: aurora kinase
CI: confidence interval
DMEM: Dulbecco's modified Eagle's medium
DMSO: dimethyl sulfoxide
DNMT: DNA methyl transferase
EGFR: epidermal growth factor receptor
EMEM: Eagle's minimum essential medium
FBS: fetal bovine serum
FGFR: fibroblast growth factor receptor
GO: gene ontology
HNSCC: head and neck squamous cell carcinoma
HPV: human papilloma virus
IC_50_
: half‐maximal inhibitory concentration
IGF‐1R: insulin‐like growth factor 1 receptor
MEV: multiple experiment viewer
PI3K: phosphatidylinositol 3‐kinase
RTK: receptor tyrosine kinase
XIAP: X‐linked inhibitor of apoptosis

## 1 INTRODUCTION

Head and neck squamous cell carcinoma (HNSCC) patients have seen limited improvement in overall survival despite advances in standard therapies such as surgery, radiation, and chemotherapy.^1^ Although the development of immune checkpoint inhibitors has improved patient outcomes and changed treatment paradigms in patients with metastatic disease, <15% of patients respond to these therapies.^2^, ^3^ Furthermore, there is a paucity of effective treatment options in patients refractory to platinum agents and immune checkpoint inhibitors. Hence, there remains a critical need to develop new therapeutic strategies including combinations of targeted agents. Given the frequency of epidermal growth factor receptor (EGFR) overexpression in HNSCC,^4^, ^5^ EGFR inhibition has been a long term target of interest for this disease. Cetuximab, an anti‐EGFR chimeric monoclonal antibody, was approved for treatment in HNSCC after demonstrating an extension in survival when combined with radiotherapy in early stage disease^6^ and cytotoxic chemotherapy in metastatic HNSCC.^7^ Nevertheless, cetuximab has limited efficacy as a single agent,^8^ and subsequent trials evaluating alternative EGFR inhibitors (including afatinib, panitumumab, and zalutumumab) have all shown similarly modest responses in HNSCC.^9^, ^10^, ^11^

Given the aforementioned successes and challenges of EGFR inhibitor therapy in HNSCC, many groups, including our own, have worked to improve EGFR inhibitor responses by developing EGFR combination approaches. For example, we and others have shown that PI3K signaling drives a common escape pathway for EGFR inhibitor resistant tumors and that combinations targeting both EGFR and PI3K are highly synergistic in HNSCC models and tumors.^12^, ^13^, ^14^, ^15^, ^16^, ^17^, ^18^, ^19^ Research in this area has also identified that inhibitors of additional receptor tyrosine kinases (RTKs) that may synergize with EGFR‐targeting agents, including inhibitors of fibroblast growth factor receptor (FGFR), insulin‐like growth factor receptor (IGF‐1R) and hepatocyte growth factor receptor (HGFR, also called MET).^20^, ^21^, ^22^, ^23^, ^24^, ^25^, ^26^, ^27^, ^28^ However, despite these discoveries, EGFR combination therapies have so far had limited clinical success.^29^, ^30^

Here, we used high throughput small molecule screening to systematically identify promising EGFR inhibitor combination strategies and characterize their effectiveness in a panel of models in order to help advance the most effective combinations that in the long term may improve outcomes in EGFR inhibitor resistant HNSCC.

## 2 MATERIALS AND METHODS

### 2.1 Cell culture

UM‐SCC cell lines were cultured in Dulbecco's Modified Eagle's Medium (DMEM) (Catalog No: 11965; Invitrogen, Carlsbad, CA) containing 10% fetal bovine serum (FBS), 1% NEAA (Catalog No: 15140122; Invitrogen, Carlsbad, CA) and 1% penicillin–streptomycin (Catalog No: 15140122; Invitrogen, Carlsbad, CA) in a humidified atmosphere of 5% CO_2_ at 37°C. HSC‐2, HSC‐4 (both from Japanese Collection of Research Bioresources through Sekisui XenoTech, Kansas City, KS) and Detroit 562 (from American Type Culture Collection, Manassas, VA) cells were cultured similarly in Eagle's Minimum Essential Medium (EMEM) (Catalog No: 30‐2003; American Type Culture Collection, Manassas, VA) with FBS and penicillin–streptomycin. Cells were genotyped to confirm authenticity and tested for mycoplasma contamination using the MycoAlert detection kit (Lonza, Basel, Switzerland).

Details of DNA copy number analysis have been described previously.^31^ All UM‐SCC cell lines were confirmed to contain wild type *EGFR* as previously reported from NimbleGen V2 exome capture based experiments.^32^

### 2.2 Chemicals

All compounds (gefitinib, BGJ398, ADW742, SGI‐1027, and BV‐6) and the inhibitor library (Table S1) were purchased from Selleck Chemicals (Houston, TX). All compounds were initially dissolved in 100% sterile DMSO to 10 mM and then diluted in media to the indicated concentrations for studies in vitro.

### 2.3 Resazurin assay

For small molecule profiling studies (both in experiments using the Selleckchem inhibitor library and in the secondary validation screen), low‐passage cell lines were frozen in large aliquots (5–10 million cells each). After aliquots were thawed, in order to eliminate drift over extended periods in culture, cells were passaged five or fewer times before fresh stocks were obtained and used. A single lot of FBS was used for all small molecule profiling and reverse‐format validation experiments. The Selleckchem inhibitor library (Table S1) was aliquoted into daughter plates, each of which was subjected to five or fewer freeze–thaw cycles before being retired from use.

Two thousand cells per well (for all cell lines except HSC‐2, for which the cell density was reduced to 1000 cells per well due to large cell size) were seeded (in 50 μl volume) in 384‐well microplates using a Multiflo liquid handling dispensing system. The following day, cells were treated with complete media containing inhibitor or DMSO using the Agilent Bravo Automated Liquid Handling Platform and VWorks Automation Control Software. For small molecule profiling studies, the Selleckchem inhibitor library (Table S1) was diluted ×20 into complete media (3 μl inhibitor into 60 μl media) and mixed well, for a final concentration of 500 μM. A second intermediate plate was generated by transferring 14 μl of 500 μM inhibitor in media to into the first quadrant of a 384 deep well plate (Catalog No: 14‐222‐227; Axygen, Union City, CA), which contained 90 μl of complete media. 30 μl from the first quadrant was transferred to the second quadrant, and 10 μl from the first quadrant was transferred to the third quadrant. For the final dilution, 30 μl of inhibitor in media from the third quadrant was added to the fourth quadrant. Following each transfer, 10 pipetting cycles were performed to ensure complete and thorough mixing of the inhibitor with the media. This intermediate plate was then diluted ×10 onto cells to achieve final drug concentrations of approximately 5, 1.5, 0.5, and 0.15 μM. Each well of cells was also treated with media containing DMSO (positive control wells and monotherapy plates) or EGFR inhibitor (combination plates) using the Multiflo liquid handling dispensing system. Gefitinib and erlotinib were both administered at 5 μM for all cell lines except UM‐SCC‐55, which was treated with EGFR inhibitors at 1 μM due to increased sensitivity to gefitinib and erlotinib as monotherapies. Data from profiling experiments will be submitted to PubChem and made publicly available.

For reverse‐format validation screens, the protocol described above for small molecule profiling was used, but the inhibitor library plate was replaced with a plate containing only DMSO (for monotherapies) and gefitinib (for combinations). After treating with DMSO or gefitinib at four concentrations, each validation inhibitor was prepared at four concentrations (2.5, 1.25, 0.625, and 0.3125 mM) in 96 well plates, diluted ×20 in complete media and then further diluted ×10 onto cells in combination with DMSO‐ or gefitinib‐treated wells. For each mono‐ and dual‐therapy in reverse‐format validation studies, treatments were performed in quadruplicate in a single 384‐well plate. For all other resazurin experiments (including secondary validation screens), cells were treated with 0.5% inhibitor or DMSO in a 10‐point two‐fold dilution series in quadruplicate. To accomplish this, 96‐well plates were prepared with inhibitors in ×200 concentration and then diluted to ×10 concentration in complete media in a second 96‐well plate using the Agilent (Santa Clara, CA) Bravo Automated Liquid Handling Platform and VWorks Automation Control Software as described previously.^13^ These inhibitors were then used to treat the cells with the desired compound concentration, again using liquid handling robotics. For combination with 5 μM gefitinib in Figure 2, 5 μl of 50 μM (×10) gefitinib in media was added to one of two 384 well plates treated in parallel with BGJ398 and ADW742 or BV‐6 combinations.

In all cases, cells were stained with 10 μl of 440 μM resazurin (Sigma, St Louis, MO) dissolved in serum‐free media for 12–24 h prior to quantification. Quantification occurred after 72‐h treatment using the Cytation3 fluorescence plate reader with 540 nm excitation and 612 nm emission wavelengths. Data were plotted to generate concentration response curves using Prism 8 software and the log(inhibitor) versus response—Variable slope model with four parameters (IC_50_, top, bottom, and Hill slope) allowed to vary.

### 2.4 Synergy scoring

We developed a scoring scheme to rank inhibitors tested as monotherapies and in combination with EGFR inhibitors based on their potentially synergistic effects in each cell line. We calculated each score (S) using the formula below, which reports the difference in relative viability between the monotherapy and combination therapy treatment at each of the four library inhibitor concentrations tested:

$$\begin{array}{rcl} S & = & \sqrt{\max\left(0,\mathrm{sign}\left(d_{1}\right)d_{1}^{2}+\mathrm{sign}\left(d_{2}\right)d_{2}^{2}+\mathrm{sign}\left(d_{3}\right)d_{3}^{2}+\mathrm{sign}\left(d\right)d_{4}^{2}\right)} \end{array}$$

where *d*_1_, *d*_2_, *d*_3_, and *d*_4_ are the absolute differences in viability following monotherapy and combination treatment for each of the four library inhibitor concentrations and sign(*d*_*i*_) is the sign (+1 for positive numbers and −1 for negative numbers) of *d*_*i*_.

After observing non‐biological responses in some of our early screening data, we added two additional qualifications to mitigate its effects and ensure that we did not overestimate the effect of any combination treatment^1^: for monotherapies, if treatment with a higher concentration of library inhibitor resulted in an unexpected higher viability, the viability was set as that of the adjacent lower concentration, and^2^ for combinations, if a lower concentration of library inhibitor resulted in an unexpected lower viability, the viability was set as that with the adjacent higher concentration. Scores <0 (which indicated that monotherapies were more effective in reducing cell viability as compared with combination treatments) were set as zero. We generated a score for each library inhibitor in combination with gefitinib and an analogous score for the library inhibitor in combination with erlotinib.

We merged synergy scores for multiple cell lines, thus evaluating the recurrence of potentially synergistic combination effects across the HNSCC models that we tested. To calculate this recurrent synergy score, EGFR inhibitor synergy scores from individual cell lines (described above) were combined in the following equation:

$$S=\;{\left(\;,\frac{\sqrt{d_{1E}}+\sqrt{d_{1G}}+\sqrt{d_{2E}}+\sqrt{d_{2G}}+\ldots +\sqrt{d_{10E}}+\sqrt{d_{10G}})}{20}\;\right)}^{2}$$

where *d*_1E_, *d*_1G_, *d*_2E_, *d*_2G_, …, *d*_10E_, and *d*_10G_ represent the combined scores for erlotinib (E) and gefitinib (G) in the 10 cell lines.

Based on our previous experience with this assay^13^, ^33^ and the degree of variability between technical replicates in an individual experiment, scores of 30 or greater were used to identify cell lines as responsive to synergistic drug combinations.

### 2.5 Heatmaps

To order responses by cell line‐EGFR inhibitor combinations, we performed hierarchical clustering with multiple experiment viewer (MEV), selecting Pearson correlation for the distance metric and average clustering for the linkage method. To generate heatmaps, we used GraphPad Prism 8 software.

### 2.6 Annexin V apoptosis assay

Cells were seeded into 6‐well plates. After 24 h, cells were treated with DMSO, monotherapy, or combination. 48 h following treatment, cells were prepared for Annexin V staining according to manufacturer recommendations (Invitrogen, Carlsbad, CA). Briefly, cells were harvested and then stained with propidium iodide and Alexa Fluor 488 annexin V before being analyzed on the Ze5 (Bio‐Rad, Hercules, CA) at the University of Michigan Flow Cytometry core.

### 2.7 Western blotting

Western blot analysis was performed as previously described.^31^, ^34^ Briefly, UM‐SCC cell lines at 70–80% confluency were rinsed with PBS and lysed in buffer with 1% NP40 containing protease and phosphatase inhibitors (Catalog Nos: 186129, 1 861 277; ThermoFisher, Waltham, MA) as described.^12^ Primary antibodies were purchased from Cell Signaling Technology (Danvers, MA) or Origene (Rockville, MD), secondary antibodies were purchased from Jackson ImmunoResearch (West Grove, PA), and catalog numbers are given in Table S2. 300 dpi or greater images were digitally retained from all westerns and representative blots are shown.

### 2.8 Mouse xenografts

Animals were housed in a vivarium accredited by the Assessment and Accreditation of Laboratory Animal Care at the University of Michigan. Veterinary care was provided by the University of Michigan Unit for Laboratory Animal Medicine, and all procedures were performed according to Institution for Animal Care and Use Committee‐approved protocol PRO00008065.

UM‐SCC‐108 cells in log‐phase growth were trypsinized and re‐suspended in a 1:1 ratio of DMEM and Matrigel (Catalog No: 354234; Corning, Corning, NY). Five‐ to 6‐week‐old, male nude athymic mice (Charles River Laboratories, Wilmington, MA) were subcutaneously injected with 2 million cells per flank. When average tumor size measured approximately 100 mm^3^, individual mice were binned so as to lessen the variance in each group. Based on previous experience with xenograft models that grow at similar rates,^32^, ^35^ eight animals (each bearing bilateral tumors) were placed in each group. We randomly assigned treatment for each group as vehicle (0.5% methylcellulose, 0.2% Tween‐80), 150 mg/kg gefitinib (maximum tolerated dose described in,^36^ 30 mg/kg BGJ398, or the combination of 150 mg/kg gefitinib and 30 mg/kg BGJ398. Treatments were delivered via oral gavage for 5 successive days, and mice were then allowed to recover for 2 days. The treatment period lasted 21 days total, during which time tumors were measured twice weekly using calipers. Tumor volume was calculated by (π/6)*(width × width × length), where length is defined by the longest measurement.^37^, ^38^ During the course of the experiment, one mouse in the combination treatment group required euthanasia, so our final analysis included seven animals (bearing 14 tumors) in this group.

Changes to cell signaling were evaluated with tumors staged to approximately 350 mm^3^ and treated using the four treatment conditions described in the previous paragraph. After 6 h, mice were humanely euthanized, and tumors were homogenized by pestle in protein lysis buffer. Western blot analysis was performed as described above.

### 2.9 Statistical analysis

To compare FITC positive cells, statistical significance was calculated on log‐transformed data fitted with linear regression and interaction term. As described previously,^13^ this test was performed using type III analysis and the ANOVA function from the “car” package in *R*. The synergy effect of the gefitinib and BGJ398 drug combination was evaluated using the *F*‐test.

Tumor volumes in UM‐SCC‐108 xenografts treated with vehicle, gefitinib, BGJ398, or combination were compared using the linear mixed model.

## 3 RESULTS

### 3.1 Small molecule profiling to identify mediators of EGFR inhibitor resistance

**FIGURE 1:**
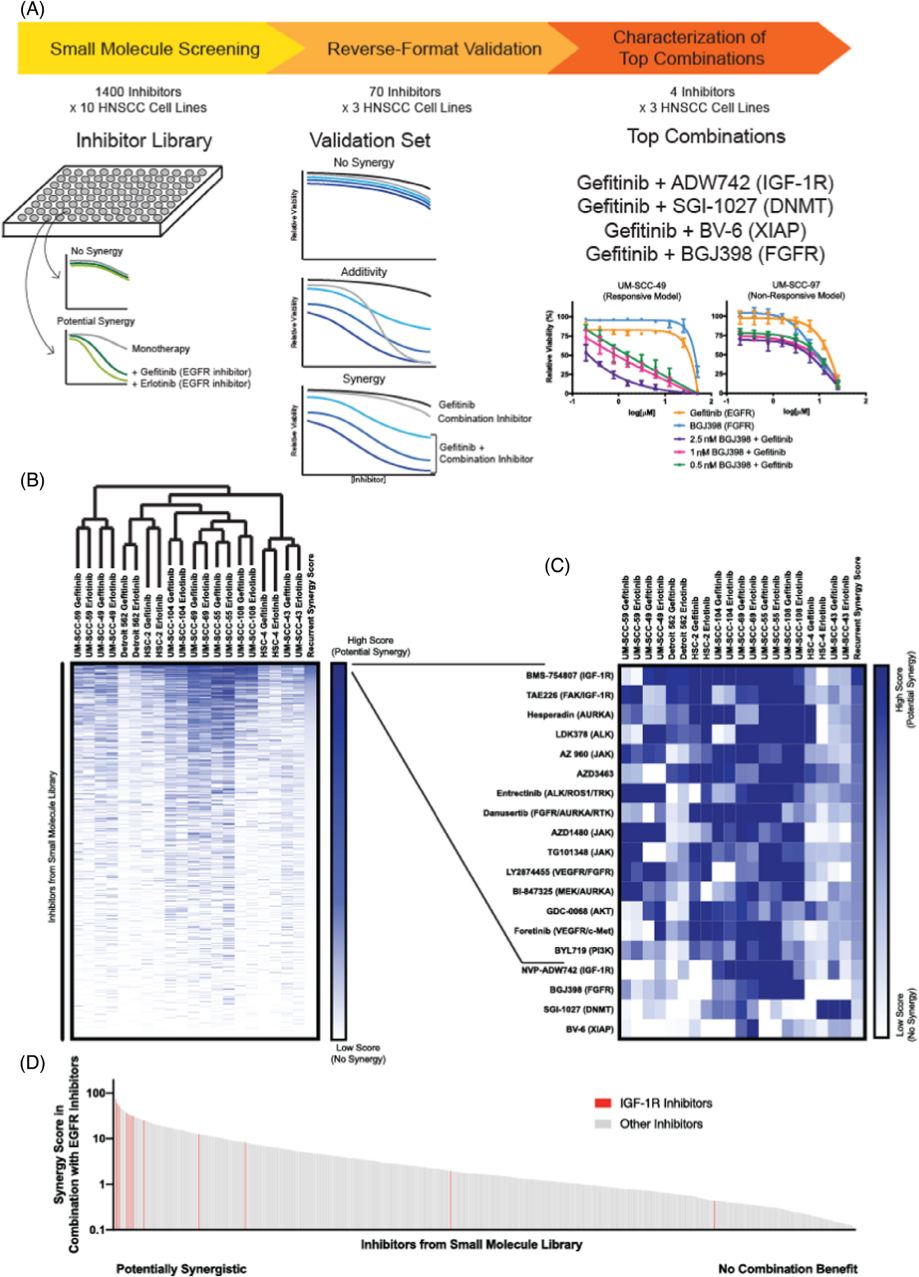
Small molecule profiling identifies synergistic epidermal growth factor receptor (EGFR) inhibitor combination treatments. (A) Strategy to identify synergistic EGFR inhibitor combinations. Small molecule profiling using a library of inhibitors (Table S1) was completed in 10 head and neck squamous cell carcinoma (HNSCC) cell lines. Validation was performed with a subset of inhibitors in three HNSCC models, and four of the most promising inhibitors were further tested in three additional cell lines. (B) Cell lines used in small molecule profiling were grouped using unsupervised hierarchical clustering based on gefitinib and erlotinib synergy scores (see Section 2 for details on scoring schemes). (C) Highlights top‐scoring combinations. (D) Recurrent synergy scores were arranged in decreasing order, and small molecule inhibitors targeting insulin‐like growth factor‐1 receptor (IGF‐1R) are shown in red. These studies were exploratory and hypothesis‐generating in nature and were validated further as described [Color figure can be viewed at wileyonlinelibrary.com]

With the hope of identifying novel dual‐therapies and deepening our understanding of the critical factors mediating EGFR inhibitor resistance in HNSCC, we utilized a small molecule profiling strategy to test a library of small molecule inhibitors as monotherapies and in combination with EGFR inhibitors (Figure 1A). For these experiments, we selected an inhibitor library that included 1406 small molecule drugs (Table S1), most of which are FDA approved or in clinical development for cancer or other diseases.^39^, ^40^, ^41^ Each inhibitor in the library was tested at four concentrations, ranging from approximately 0.15 to 5 μM, and was applied both as a monotherapy and in combination with gefitinib or erlotinib. EGFR inhibitors were added at micromolar concentrations, which had little effect in reducing cell viability but were sufficient to block EGFR phosphorylation at the tyrosine 1068 residue. Combinatorial screening was performed in 10 HNSCC cell lines, including one human papillomavirus (HPV) positive (UM‐SCC‐104) and nine HPV negative models. All cell lines tested harbored wild type EGFR, and most displayed high‐level amplification of this gene with up to 23 copies (UM‐SCC‐59).^31^

In all, our exploratory efforts evaluated nearly 3000 EGFR inhibitor dual‐therapies in each of the 10 HNSCC models and generated a combined dataset with more than 150 000 data points. To assess the effects of each EGFR inhibitor combination, we generated “synergy scores” for the effects of each inhibitor in combination with gefitinib (“gefitinib score”) and with erlotinib (“erlotinib score”). Hierarchical clustering of synergy scores for each set of drug‐cell line combinations grouped responses to gefitinib and erlotinib combinations for each HNSCC model, demonstrating that common combinatorial effects were observed with multiple means of EGFR inhibition (Figure 1B).

To identify combinations with recurrent effects in multiple models, we combined the gefitinib and erlotinib scores for each agent across the 10 cell lines; this metric was termed as the “recurrent synergy score.” Of the inhibitors tested, the agents with the 15 highest recurrent synergy scores included several with activity against IGF‐1R (BMS‐754807, TAE226, AZD3463), FGFR (danusertib, LY2874455), and PI3K or downstream target AKT (GDC‐0068, BYL719, and GSK1059615) (Figure 1C). IGF‐1R targeting agents in combination with gefitinib and erlotinib were particularly effective in these models, clustering among those agents with the highest recurrent synergy scores (Figure 1D).^26^ For example, 9/10 (90%) cell lines tested were responsive to BMS‐754807 with erlotinib and/or gefitinib. Similarly, all but one model was responsive to combination of EGFR inhibitor and FGFR inhibitor LY2874455 and 8/10 (80%) models were sensitive to gefitinib and/or erlotinib with danusertib. Other combinations with potentially synergistic effects included anaplastic lymphoma kinase (ALK) and aurora kinase (AURKA) inhibitors, as previously reported.^42^, ^43^, ^44^, ^45^ Three JAK inhibitors were also among our list of compounds with potential synergy in combination with gefitinib and/or erlotinib, consistent with demonstrations of JAK/STAT signaling as a mechanism of resistance to EGFR targeted therapy in non‐small cell lung cancer.^46^, ^47^ Thus, we rediscovered multiple mechanisms of EGFR inhibitor resistance that have been independently confirmed in preclinical models and demonstrated the validity of our approach.

Next, in order to confirm the combination effects of specific drug pairs, we used our synergy scores to nominate ~5% of the inhibitors from our small molecule library for additional testing using a larger number of dose combinations in a reverse format. Here, gefitinib was titrated into four constant concentrations of each of the validation inhibitors, and effects on cell viability were compared with the efficacy of the gefitinib and validation inhibitors as monotherapies (Figure 1A). In this way, we inverted the method used in the initial small molecule profiling experiments to perform validation screening in three cell lines with EGFR amplification (HSC‐2, HSC‐4, and Detroit 562). Following the completion of these studies, we manually placed the validation inhibitors into groups with^1^ little to no efficacy alone or in combination with gefitinib,^2^ additive effects in combination with gefitinib, or^3^ potentially synergistic effects in combination with gefitinib. Inhibitors in the latter group included many targets discussed above, including IGF‐1R, FGFR, PI3K, AKT, ALK, and AURKA. We also observed potential synergy with combinations of gefitinib and XIAP inhibitor BV‐6 and DNMT inhibitor SGI‐1027, agents that had shown potential synergy with gefitinib and/or erlotinib in three and four models, respectively, in the original screen.

### 3.2 Characterization of response to EGFR inhibitor combinations

**FIGURE 2:**
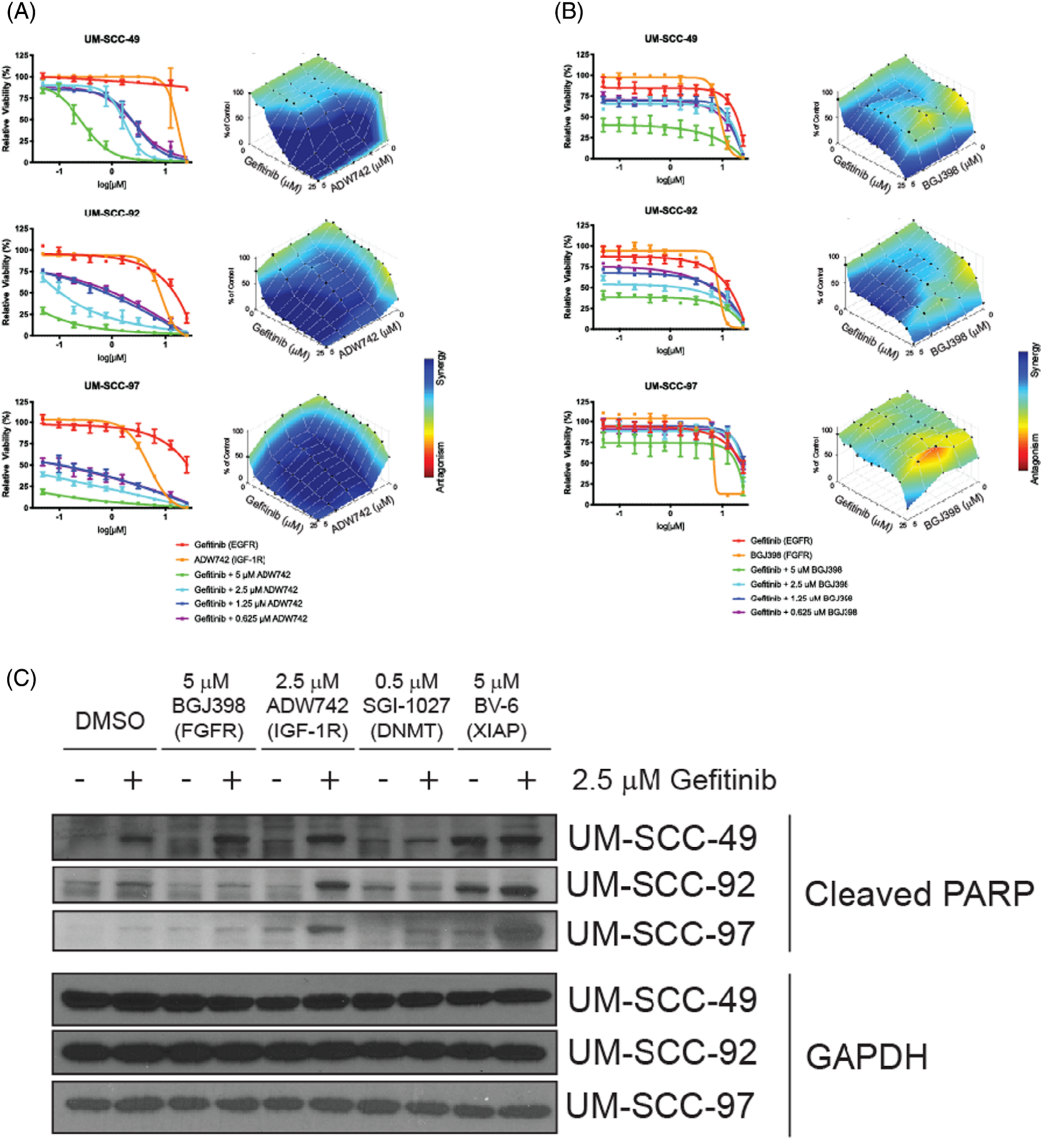
Effect of epidermal growth factor receptor (EGFR) Inhibitor dual‐therapies on cell viability, apoptosis, and downstream signaling. UM‐SCC‐49, −92, and −97 cells were treated with increasing concentrations of EGFR inhibitor gefitinib and/or insulin‐like growth factor‐1 receptor (IGF‐1R) inhibitor ADW742 (A) or fibroblast growth factor receptors (FGFR) inhibitor BGJ398 (B) for 72 h. Cell viability was measured using a resazurin cell viability assay. Each point is the mean and SD of quadruplicate determinations from a single experiment. Each experiment was repeated independently at least twice with similar combination effects based on previous experience with these assays^13^; representative data is shown along with analysis using Combenefit software.^60^ (C) Western blot analysis of PARP cleavage following 24‐h treatment with DMSO, 2.5 μM ADW742, 5 μM BGJ398, 0.5 μM DNA methyl transferase (DNMT) inhibitor SGI‐1027, or 5 μM X‐linked inhibitor of apoptosis (XIAP) inhibitor BV‐6 in the presence or absence of 2.5 μM gefitinib. GAPDH was used as a loading control. Representative images from at least two independent experiments are shown [Color figure can be viewed at wileyonlinelibrary.com]

Based on these findings, we set out to compare the effects of gefitinib in combination with IGF‐1R inhibitor ADW742, FGFR inhibitor BGJ398, BV‐6, or SGI‐1027 in three additional HNSCC models (UM‐SCC‐49, −92, and − 97). We first performed high‐density resazurin cell viability assays to assess synergy in these cell lines after 72‐h treatment, observing substantial combination benefit in all three following co‐treatment with gefitinib and ADW742 (Figure 2A). UM‐SCC‐49 and UM‐SCC‐92 also responded synergistically to EGFR and FGFR inhibition, while UM‐SCC‐97 was not responsive to the gefitinib and BGJ398 combination (Figure 2B). Combining gefitinib with high concentrations of BV‐6 was synergistic in UM‐SCC‐92 and UM‐SCC‐97, while SGI‐1027 was more effective as a monotherapy but did little to shift responses to gefitinib (Figure S1). We also explored the ability of 24‐h mono‐ and dual‐therapy treatment with these combinations to induce apoptosis, as measured using PARP cleavage. Consistent with our viability data, treating each of the three cell lines with 2.5 μM gefitinib and 2.5 μM ADW742 resulted in increased expression of cleaved PARP. On the other hand, responses to gefitinib and 5 μM BGJ398 were less robust at this time point (Figure 2C). However, EGFR and FGFR inhibitor combination treatments do synergistically increase annexin V positivity after 48‐h treatment in UM‐SCC‐49 cells and have lesser effects when used in the UM‐SCC‐97 model (see Figure 4C), thereby again corroborating with viability data. Finally, gefitinib and 0.5 μM SGI‐1027 did not result in the induction of cleaved PARP, and the gefitinib and BV‐6 combination increased cleaved PARP expression only in UM‐SCC‐97 cells (Figure 2C).

**FIGURE 3:**
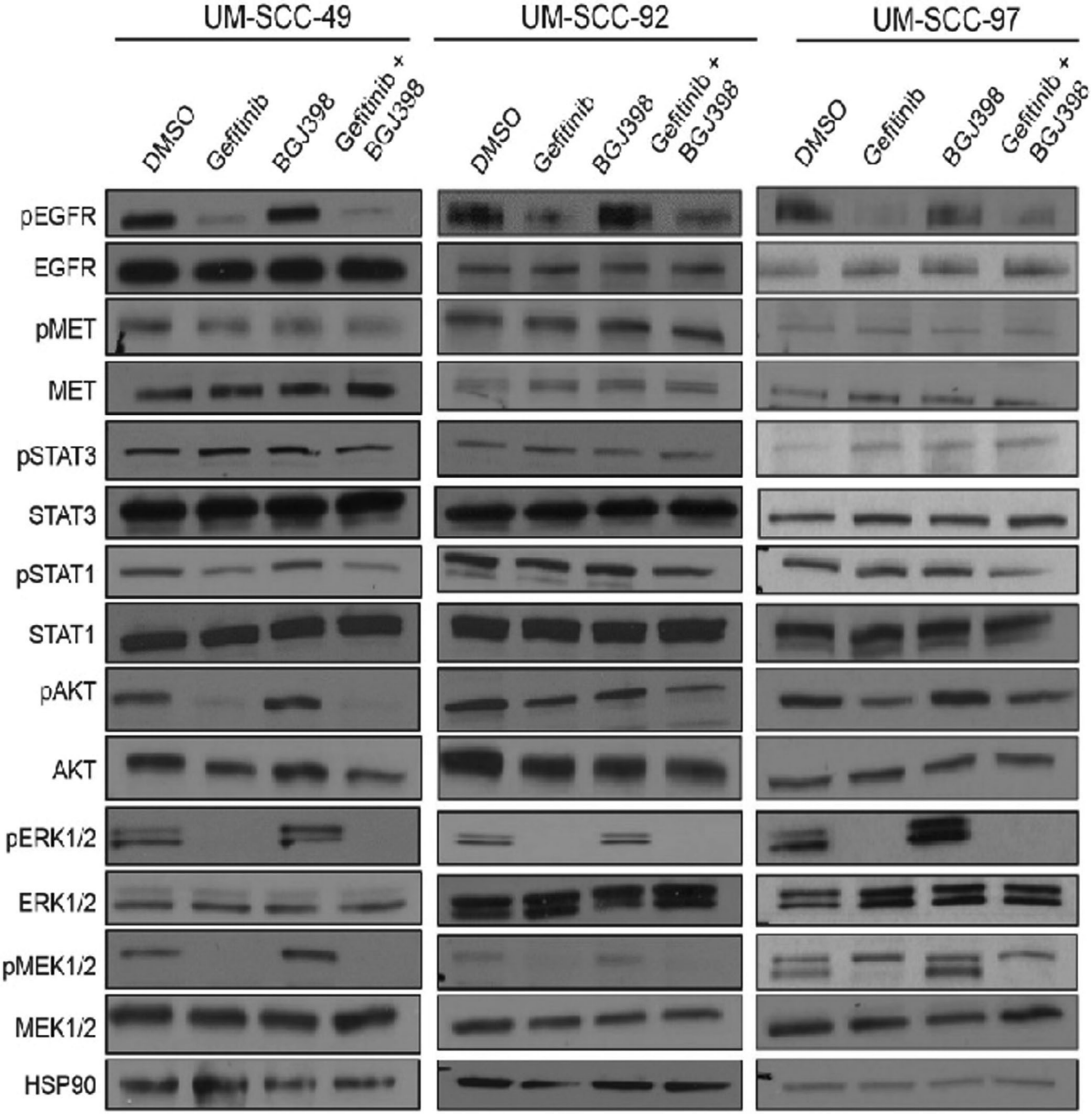
Response of signal transduction pathways to combination therapy in synergistic and non‐synergistic models. Western blot analysis of phosphorylated and total EGFR, STAT3, STAT1, AKT, ERK, and MEK expression following 1‐h treatment with DMSO, 1 μM gefitinib, 1 μM BGJ398, or combination in UM‐SCC‐49, −92, and −97 cells. HSP90 was used as a loading control. Experiments were performed in duplicate based on previous experience with these assays, and representative images are shown. All analysis steps had been decided before we looked at the data

We then interrogated the changes in signaling that follow combination EGFR inhibitor treatment in responsive and non‐responsive models by examining responses downstream of EGFR in each of the UM‐SCC‐49, −92, and −97 cell lines. To do so, we treated cells with vehicle DMSO, 1 μM gefitinib, 1 μM BGJ398, or the combination for 1 h and harvested the cell lysates for western blot analysis. We hypothesized that maintained phosphorylation of a downstream effector might explain the lack of synergy following EGFR and FGFR inhibition in non‐responsive UM‐SCC‐97 cells. However, in each of the models tested, including UM‐SCC‐97, the phosphorylation of AKT, ERK1/2, and MEK1/2 decreased in response to the EGFR and FGFR inhibitor dual‐therapy (Figure 3). We also noted limited changes in phosphorylation of STAT1 or STAT3 across treatments, suggesting that the JAK/STAT pathway has a limited role in response to gefitinib and BGJ398 combinations. Notably, the phosphorylation of MET, another known resistance pathway to EGFR inhibition,^27^, ^28^ did not change in response to EGFR inhibition for either of the models that respond to the EGFR and FGFR inhibitor combination or for UM‐SCC‐97. The lack of induction in MET phosphorylation in combination responses may indicate that MET is not a compensatory pathway in these models. Overall, we did not observe any distinguishing factor that differentiated a responsive cell line model from a non‐responsive model.

**FIGURE 4:**
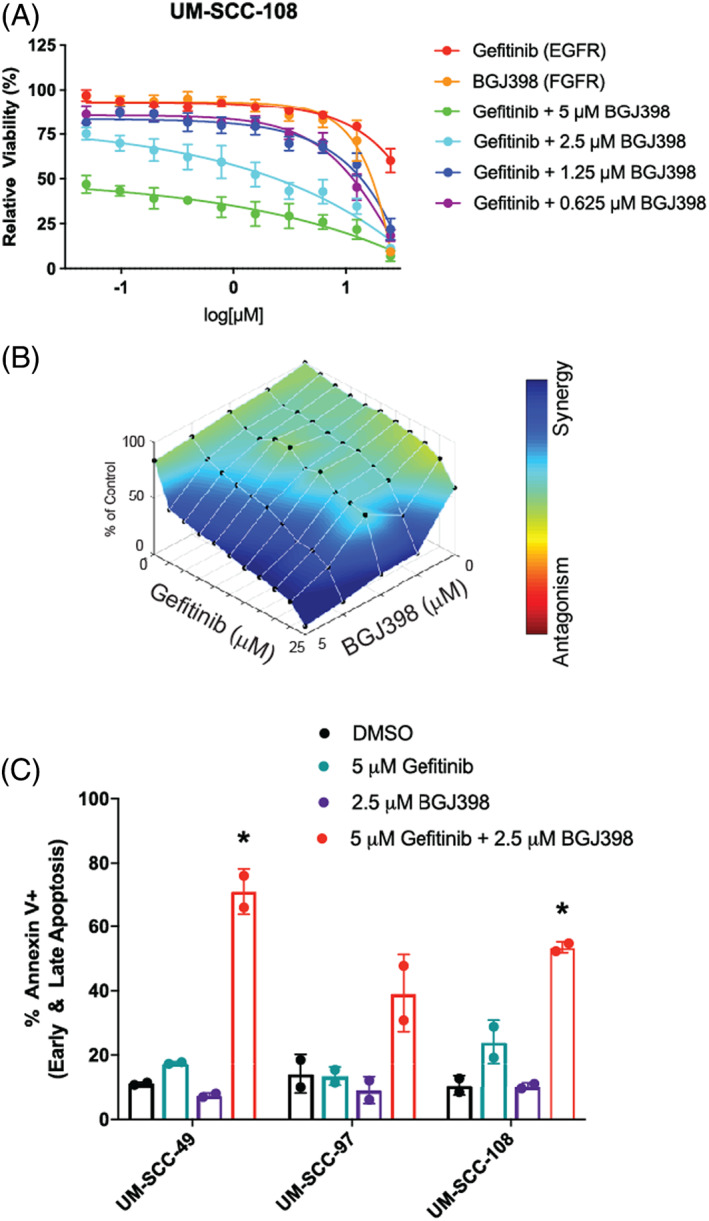
Response of UM‐SCC‐108, a model that grows well in mice, to epidermal growth factor receptor (EGFR) and fibroblast growth factor receptor (FGFR) inhibitor combination. (A) UM‐SCC‐108 cells were treated with increasing concentrations of EGFR inhibitor gefitinib and/or FGFR inhibitor BGJ398 (B) for 72 h. Cell viability was measured using a resazurin cell viability assay. Each point is the mean and SD of quadruplicate determinations from a single experiment. Each experiment was repeated independently at least twice with similar combination effects based on previous experience with these assays^13^; (B) representative data is shown along with analysis using Combenefit software.^60^ (C) Combination responsive models UM‐SCC‐49 and ‐108 and combination nonresponsive models UM‐SCC‐97 were treated with DMSO, 5 μM EGFR inhibitor gefitinib, 2.5 μM FGFR inhibitor BGJ398, or both gefitinib and BGJ398 for 48 h. The percentage of annexin V positive cells was measured after cells were stained with FITC and PI using an annexin V apoptosis assay. Scatter plot shown represents two independent experiments with bars showing the mean percentage of annexin V positive cells based on previous experience with these assays.^13^ * indicates significance with *p* < 0.05 using two‐way analysis of variance (ANOVA), as described above in Section 2 [Color figure can be viewed at wileyonlinelibrary.com]

We next wanted to study the effects of EGFR inhibition in vivo and further validate the FGFR inhibitor combination identified in our small molecule screen. As UM‐SCC‐49, −92, and −97 cells do not reliably form flank xenografts, we selected another model that was sensitive to the EGFR and FGFR inhibitor dual‐therapy based on our previous screening data and that was also capable of forming xenografts in mice. Thus, using UM‐SCC‐108 cells, we first validated the synergistic ability of gefitinib and BGJ398 to reduce cell viability using a resazurin cell viability assay in vitro (Figure 4A,B). Furthermore, we determined that UM‐SCC‐108 cells undergo apoptosis following 48‐h treatment with gefitinib and BGJ398. Following combination treatment, UM‐SCC‐108 cells display higher levels of annexin V positivity than cells treated with either monotherapy (*p* = 0.015, two‐way ANOVA, Figure 4C); these effects mirrored those observed in UM‐SCC‐49, but not UM‐SCC‐97 cells (*p* = 0.021 and 0.31, respectively, two‐way ANOVA).

**FIGURE 5:**
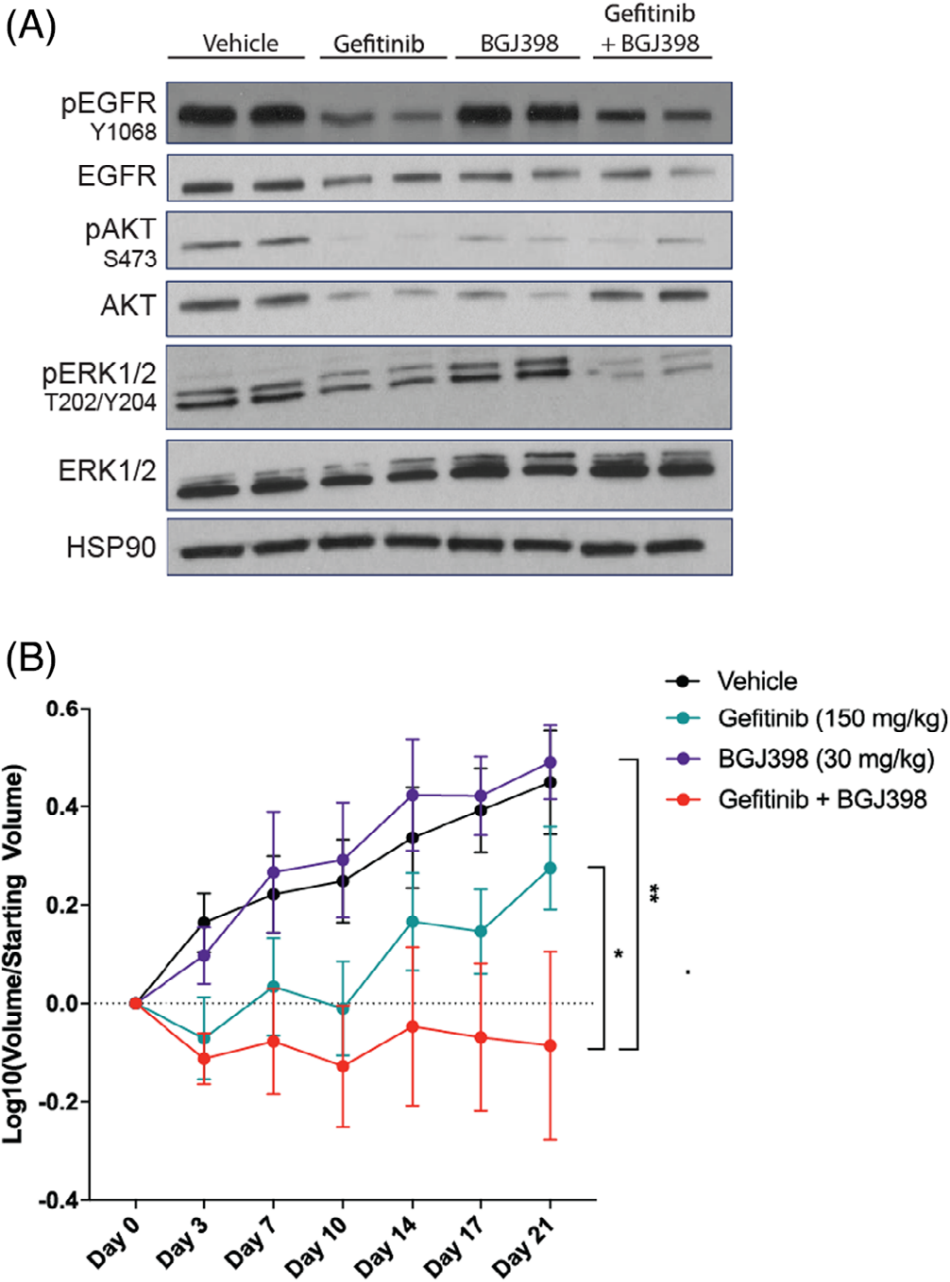
UM‐SCC‐108 is sensitive to combined epidermal growth factor receptor (EGFR) and fibroblast growth factor receptor (FGFR) inhibition in vivo. Two million UM‐SCC‐108 cells were injected into each flank of athymic nude mice. Mice were treated with vehicle, 150 mg/kg gefitinib, 30 mg/kg BGJ398, or combination. (A) Following a single 6‐h treatment, mice were humanely euthanized and tumors were harvested. Western blot analysis was performed for indicated proteins, and HSP90 was used as a loading control. Over the course of 3 weeks of treatment (five treatments/week), (B) tumor volume (mean ±95% CI) were recorded for *n* = 7–8 based on previous experience with similar xenografts^32^, ^35^ [Color figure can be viewed at wileyonlinelibrary.com]

Having shown that the UM‐SCC‐108 model responds to EGFR and FGFR inhibitor in vitro, we established UM‐SCC‐108 xenografts and began treatment with vehicle, 150 mg/kg gefitinib monotherapy, 30 mg/kg BGJ398 monotherapy, or gefitinib and BGJ398 combination. Pharmacodynamic analyses in tumors staged to 100 mm^3^ indicated that 6‐h treatment with gefitinib monotherapy or combination reduced EGFR phosphorylation at the tyrosine 1068 residue. Gefitinib monotherapy was also capable of blocking ERK phosphorylation, and both monotherapies led to decreased phosphorylation of AKT. Combination treatment resulted in a further reduction of AKT and ERK phosphorylation as compared with vehicle or monotherapies (Figure 5A). We then assessed the result of long‐term treatments on tumor growth. After therapies were administered five times per week for 3 weeks, we observed that tumor volume was reduced in mice bearing xenografts treated with gefitinib (*p* = 0.055, linear mixed model) and reduced significantly further with gefitinib and BGJ398 dual‐therapy treatment (*p* = 0.0011 and 0.011 vs. vehicle and gefitinib monotherapy, respectively, linear mixed model) (Figure 5B), thus confirming the substantial effects we had seen in our cell culture models. Mice receiving vehicle or monotherapy treatments increased in weight over the course of the experiment, while mice treated with the combination did not gain weight over the course of the experiment (Figure S2). These results are critical as they demonstrate the ability of our approach to identify combinations capable of reducing tumor burden in animal models and suggest that clinically viable EGFR and FGFR inhibitor combinations such as lenvatinib and gefitinib^48^ warrant further detailed pharmacokinetic characterization in HNSCC.

## 4 DISCUSSION

The primary purpose of our study was to validate a high throughput drug screening platform for the discovery of synergistic EGFR inhibitor combinations, and our approach identified several highly effective EGFR inhibitor combinations in our panel of models. For example, while none of the UM‐SCC models that we tested were sensitive to either FGFR or EGFR inhibition as monotherapies, our results were consistent with the literature showing a strong synergy of the combination and supporting the ability of our approach to re‐discover synergistic combinations.^22^, ^23^ Further, our profiling experiment demonstrates that FGFR signaling may be a more common compensatory mechanism in cells treated with EGFR inhibitors than previously realized. While some work on the dual inhibition of EGFR and FGFR has already been accomplished preclinically,^22^, ^23^ the most limiting factor in translating this combination to the clinic has been the toxicity of combining EGFR and FGFR inhibitors. For example, a trial of the EGFR inhibitor erlotinib and pan‐FGFR inhibitor dovitinib in metastatic non‐small cell lung cancer was halted early given dose limiting toxicities.^30^ Thus, in the future, it is important to identify alternative and less cytotoxic approaches to inhibit the downstream effectors of this common resistance pathway. Indeed, data already suggest that newer and less toxic treatments targeting EGFR and FGFR may well‐tolerated such as the combination of gefitinib and levatinib that was recently evaluated in liver cancer (NCT04642547).^48^ These data, as well as our own, show that future work evaluating more specific inhibitors (including PROTACs) and/or the pivotal effectors downstream of the response to EGFR and FGFR inhibitor combinations may identify appropriate combinations for clinical advancement.

Our small molecule profiling strategy also led us to re‐discover the strong synergistic effects of EGFR and IGF‐1R inhibition. Previous work has examined cross‐talk between IGF‐1R and EGFR signaling in HNSCC to show that IGF‐1R activation leads to EGFR inhibitor resistance^26^ and IGF‐1R inhibition results in compensatory activation of EGFR.^49^ Despite the established preclinical rationale for combining EGFR and IGF‐1R inhibition in HNSCC, few clinical trials evaluating the simultaneous inhibition of these pathways have been completed. One of the only reported studies (NCT0617734) compared the effects of IMC‐A12, a monoclonal antibody against IGF‐1R, when administered as a single agent or in combination with cetuximab. IMC‐A12 mono‐ and dual‐therapies with EGFR inhibition, however, did not differ significantly in their effects on patient outcomes. As such, these results again support the need for further work assessing the molecular mechanisms of EGFR inhibitor resistance in HNSCC and translating these data to the clinic. Our data highlight cell lines, such as UM‐SCC‐59, that can be used to model such resistance to combined EGFR and IGF‐1R inhibition. These studies may aid in the identification of resistance mechanisms and response biomarkers.

Our screen also identified several novel EGFR inhibitor combinations with synergy in our models, including DNMT and XIAP inhibitors. Immunohistochemical studies and gene expression arrays have previously demonstrated high expression of DNMTs in many oral cancers.^50^, ^51^ Furthermore, DNMT1 overexpression is associated with reduced time to relapse, and clones with high DNMT3B expression display an invasive phenotype.^50^, ^52^ The relationship between EGFR signaling and DNA methylation, however, is still somewhat understudied in both HNSCC and other cancer types. Samudio‐Ruiz and Hudson previously showed that EGFR activation in ovarian cancer leads to a pattern of global methylation, reversible upon treatment with a hypomethylating agent.^53^ In our HNSCC cell lines, gene amplification and/or protein overexpression of EGFR may be responsible for similar epigenetic changes and could serve to silence important tumor suppressor genes. Adding DNMT inhibition to EGFR inhibitor targeted therapy may thereby restore the function of genes facilitating apoptosis following drug treatment. As such, further work is needed to better elucidate the mechanism underlying synergistic effects of gefitinib and SGI‐1027 in some models.

*XIAP* expression has also been described as an early and important event in oral carcinogenesis,^54^ and XIAP antagonists are known to improve treatment responses in HNSCC when combined with several other therapies.^55^, ^56^, ^57^ Some work has also shown that XIAP inhibition can sensitize to EGFR targeting agents. For example, in non‐small cell lung cancer models, XIAP antagonist HM90822B was effective as a monotherapy, particularly in cases with activated EGFR signaling.^58^ Furthermore, study by Foster et al. used XIAP siRNA to sensitize breast cancer cells to gefitinib treatment, and their work also suggested that inhibition of another ERBB family member, HER2, also can cooperate with XIAP siRNA^59^; as such, it will be informative to explore the role of both EGFR and other ERBB family members in synergistic responses. Indeed, with the recent emergence of XIAP inhibitors in the clinic, these combination therapies will certainly warrant further evaluation in future HNSCC studies.

Collectively, our data highlight the value of combinatorial drug screening for both the identification of synergistic combinations as well as to help understand the effectiveness of various combinations across a panel of models with high EGFR expression. Future studies could expand on this screening approach to investigate both of these important questions and long‐term the approach may lead to the identification of effective drug combination that lead to clinical benefit in HNSCC patients.

## AUTHOR CONTRIBUTIONS

Participated in research design: Michmerhuizen, Ludwig, Bradford, Swiecicki, Carey, Brenner. Conducted experiments: Michmerhuizen, Ludwig, Birkeland, Nimmagadda, Wang, Jewell, Genouw, Remer, Kim, Foltin. Contributed new reagents or analytic tools: Ludwig, Birkeland, Nimmagadda. Performed data analysis: Michmerhuizen, Ludwig, Zhai, Bhangale, Kulkarni, Jiang, Brenner. Wrote or contributed to the writing of the manuscript: Michmerhuizen, Ludwig, Brenner.

## ETHICS AND INSTITUTIONAL APPROVAL

Experimental animal approvals were obtained for our institutional animal review board, and no human subjects were used for the research.

## Supporting information


**FIGURE S1** Effect of prioritized combinations on UM‐SCC cell proliferation. UM‐SCC‐49, −92, and −97 cells were treated with increasing concentrations of EGFR inhibitor gefitinib and/or DNMT inhibitor SGI‐1027 (A) or XIAP inhibitor (B) for 72 h. Cell viability was measured using a resazurin cell viability assay. Each point is the mean and SD of quadruplicate determinations from a single experiment. Each experiment was repeated independently at least twice with similar combination effects: representative data is shown along with analysis using Combenefit software^60^

**FIGURE S2** Effect of combination EGFR and FGFR inhibitor therapy on mouse weight. Mice treated in Figure 5 were weighed twice weekly.

**TABLE S1** List of drugs, drug targets and pathway targets within the drug screening library.

**TABLE S2** Primary antibodies used in this study for Western blotting.

## Data Availability

The data that support the findings of this study are available from the corresponding author upon reasonable request.
