# Supplementary figures and images for: Small molecule profiling to define synergistic EGFR inhibitor combinations in head and neck squamous cell carcinoma
Source: Head Neck. 2022 Feb 27;44(5):1192–205. doi: 10.1002/hed.27018 (PMC8986607; doi:10.1002/hed.27018)

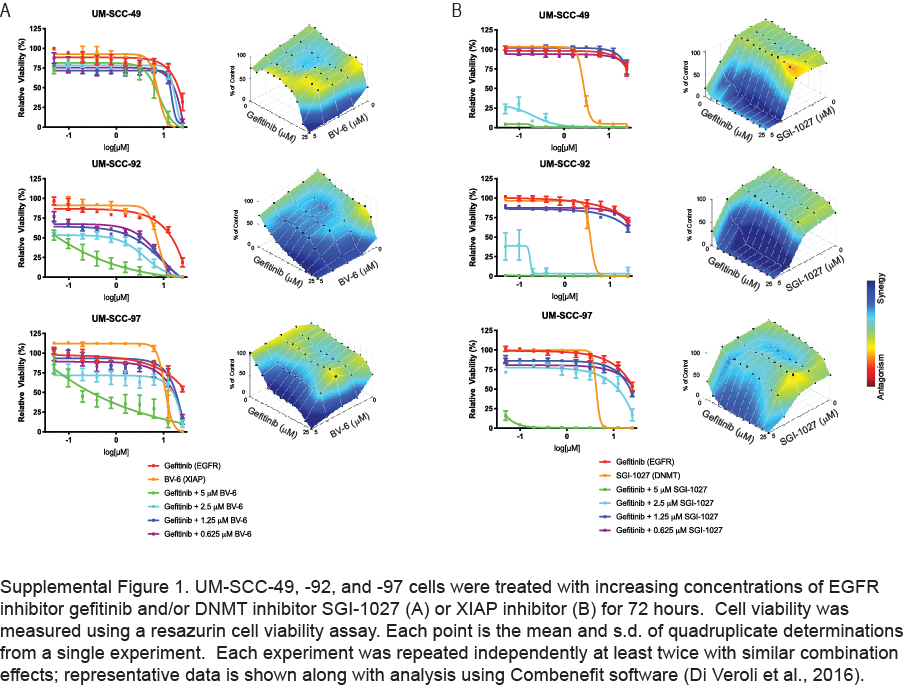

Supplement: Supplementary file 1 — FIGURE S1 Effect of prioritized combinations on UM‐SCC cell proliferation. UM‐SCC‐49, −92, and −97 cells were treated with increasing concentrations of EGFR inhibitor gefitinib and/or DNMT inhibitor SGI‐1027 (A) or XIAP inhibitor (B) for 72 h. Cell viability was measured using a resazurin cell viability assay. Each point is the mean and SD of quadruplicate determinations from a single experiment. Each experiment was repeated independently at least twice with similar combination effects: representative data is shown along with analysis using Combenefit software 60 [file HED-44-1192-s002.docx]

## Slide 1
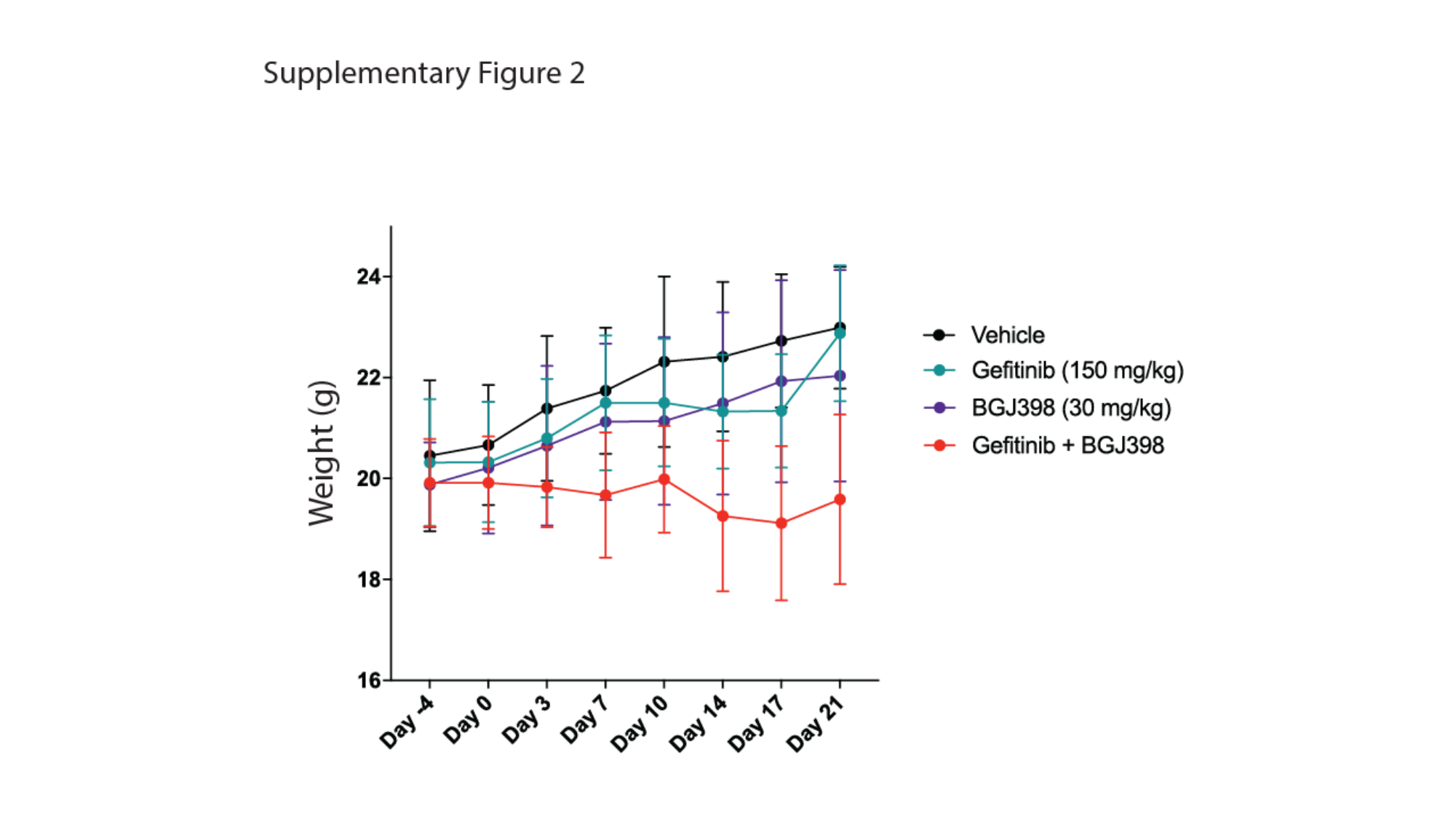

Supplement: Supplementary file 2 — FIGURE S2 Effect of combination EGFR and FGFR inhibitor therapy on mouse weight. Mice treated in Figure 5 were weighed twice weekly. [file HED-44-1192-s001.pptx]
