# Supplementary material for: Small molecule profiling to define synergistic EGFR inhibitor combinations in head and neck squamous cell carcinoma
Source: Head Neck. 2022 Feb 27;44(5):1192–205. doi: 10.1002/hed.27018 (PMC8986607; doi:10.1002/hed.27018)
Supplement: Supplementary file 4 — TABLE S2 Primary antibodies used in this study for Western blotting. [file HED-44-1192-s004.docx]

**Table S2. Primary Antibodies for Western Blotting**

| **Antibody** | **Catalog #** |
| --- | --- |
| p-EGFR Y1068 | CST 3777 |
| EGFR | CST 8504 |
| p-AKT S473 | CST 4060 |
| AKT | CST 4685 |
| pERK1/2 T202/Y204 | CST 4370 |
| ERK1/2 | CST 4695 |
| p-MEK1/2 S217/221 | CST 9121 |
| MEK1/2 | CST 8727 |
| Cleaved PARP | CST 5625 |
| p-STAT1 S727 | CST 8826 |
| STAT1 | CST 14994 |
| p-MET Y1234/1235 | CST 3077 |
| MET | CST 8198 |
| p-STAT3 Y705 | CST 9145 |
| STAT3 | CST 30835 |
| HSP90 | CST 4877 |
| GAPDH | CST 5174 |
| Anti-Rabbit Secondary | Jackson Research 111-035-045 |
| Anti-Mouse Secondary | Jackson Research 715-035-151 |
